# The Effect of Evogliptin Tartrate on Controlling Inflammatory Pain

**DOI:** 10.3390/biomedicines11112990

**Published:** 2023-11-07

**Authors:** Pyung Goo Cho, Jun Ho Jang, Sukjin Ko, Dong Ah Shin, Seungsoo Chung, Min Cheol Chang

**Affiliations:** 1 Department of Neurosurgery, Ajou University Medical Center, Suwon-si 16499, Republic of Korea; nsdrcpg@gmail.com; 2 BnH Research Co., Ltd., Goyang-si 10594, Gyeonggi-do, Republic of Korea; talktojunho@gmail.com; 3 Department of Physiology, Graduate School of Medical Science, Brain Korea 21 Project, Yonsei University College of Medicine, Seoul 03722, Republic of Korea; zkingzko@naver.com; 4 Department of Neurosurgery, Yonsei University College of Medicine, Seoul 03722, Republic of Korea; cistern@yuhs.ac; 5 Department of Physical Medicine & Rehabilitation, College of Medicine, Yeungnam University, Daegu 42415, Republic of Korea

**Keywords:** evogliptin tartrate, inflammation, pain, medication, cytokine, resting membrane potential, action potential

## Abstract

**Background**: Evogliptin tartrate inhibits dipeptidyl peptidase-4 (DPP-4), boosting glucagon-like peptide 1 (GLP-1) secretion and improving insulin release and glucose tolerance, while also exerting anti-inflammatory effects. We investigated its anti-inflammatory and analgesic effects. **Methods**: Forty male Sprague Dawley rats were divided into (N = 10 in each): (1) naïve, (2) complete Freund’s adjuvant (CFA) inflammation + evogliptin tartrate (once for 10 mg/kg) (CFAE), (3) CFA + vehicle (same volume with normal saline with evogliptin tartrate/once) (CFAV), and (4) CFA + indomethacin (5 mg/mL/kg/1 time) (CFAI) groups. CFA was injected subcutaneously into rat plantar regions, and medications (evogliptin tartrate, vehicle, and indomethacin) were administered orally for 5 days. Post treatment, blood from the heart and plantar inflammatory tissue were collected to assess inflammatory cytokines. Evogliptin tartrate effects on controlling inflammation and pain were evaluated by measuring rat plantar paw thickness, paw withdrawal threshold, dorsal root ganglion (DRG) resting membrane potential, DRG action potential firing, and cytokine (TNF-α and IL-1β) levels. **Results**: Compared with the naïve group, plantar paw thickness, cytokine (TNF-α and IL-1β) levels, DRG resting membrane potential, and DRG action potential firing increased, whereas the paw withdrawal threshold decreased in all CFA groups. However, CFAE and CFAI rats showed recovery. The degree of CFAE recovery resembled that observed in the CFAI group. **Conclusions**: Evogliptin tartrate mirrored the anti-inflammatory pain relief of indomethacin. We aim to broaden its use as an anti-inflammatory drug or pain relief drug.

## 1. Introduction

Inflammatory pain is caused by peripheral tissue injury and inflammation [1]. The perception of an effective response to noxious stimuli occurs during an inflammatory or immune response [1]. An inflammatory response is a complex sequence of physiological processes that occurs following an injury or infection, aimed at combating and resolving the associated conditions [2]. Inflammation is marked by five distinct symptoms: localized redness, increased temperature, swelling, pain, heightened sensitivity, and loss of function [3]. Inflammation is a crucial protective mechanism that is necessary for wound healing [4]. However, acute inflammation induces pronounced pain by directly stimulating nociceptive neurons in the inflamed tissue [5]. Non-steroidal anti-inflammatory drugs (NSAIDs) manage inflammatory pain by impeding the synthesis of prostaglandins and thromboxanes through the inhibition of cyclooxygenase [6]. Nevertheless, NSAIDs are associated with several adverse effects on the gastric mucosa and the cardiovascular, renal, hepatic, and hematologic systems [6].

Recently, evogliptin (DA-1229) tartrate was developed to control blood glucose levels in patients with type 2 diabetes [7]. Chemically, it is known as (3R)-4-[(3R)-3-amino-4-(2,4,5-trifluorophenyl) butanoyl]-3-[(2-methylpropan-2-yl) oxymethyl] piperazin-2-one or (2R,3R)-2,3-dihydroxybutanedioic acid [8]. It functions by inhibiting dipeptidyl peptidase-4 (DPP-4) and enhancing the secretion of incretin hormones, including glucagon-like peptide-1 (GLP-1) [9,10,11]. Elevated GLP-1 levels contribute to the reduced production of various inflammatory cytokines [9,10,11]. Consequently, we propose that evogliptin tartrate could potentially aid in the management of inflammatory pain.

The present study aimed to elucidate the anti-inflammatory effects of evogliptin tartrate and investigated its potential in controlling inflammatory pain. We sought to explore the feasibility of extending the application of evogliptin tartrate, originally developed for controlling blood glucose levels in patients with type 2 diabetes, as a viable option for mitigating inflammation or pain induced by inflammation.

## 2. Methods

### 2.1. Animal Model

In our experiments, we utilized 40 adult male Sprague Dawley rats (aged 5–6 weeks), which were purchased from Deahan biolink Co., Chungcheongbuk-do, Republic of Korea. All procedures were performed in accordance with the protocols approved by the Institutional Animal Care and Use Committee of the Yonsei University Health System on 1 November 2018 (approval code: 2018-0272).

### 2.2. Preparation of Pain Animal Model and Administration of Experimental Drug

The rats were housed in a laboratory animal facility throughout the duration of the experiment. They had access to food and water ad libitum, and were maintained under controlled conditions of constant temperature (22 ± 1 °C) and relative humidity (50 ± 10%). These rats were subjected to a 12 h light–dark cycle each day, alternating between periods of light and darkness.

**Figure 1:**
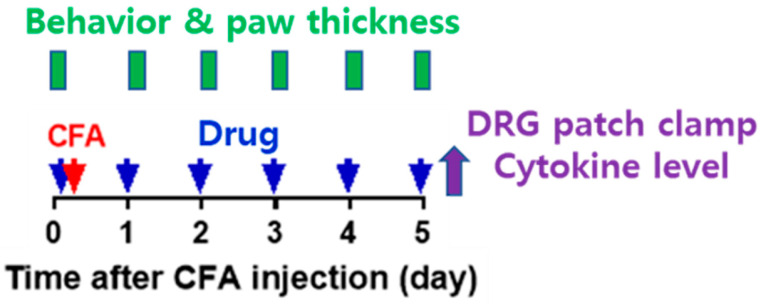
Schematic of the experiment schedule (CFA: complete Freund’s adjuvant, DRG: dorsal root ganglia, red arrow: completion of CFA inflammation model, blue arrows: administration of drugs, purple arrow: experiments for DRG patch clamp and cytokine level).

Forty rats were randomly assigned to one of four experimental groups after a 1-week adaptation period. A total of 10 rats were allocated to each of the four groups: (1) a naïve group, (2) a complete Freund’s adjuvant (CFA) inflammation model + evogliptin tartrate (Suganon^®^, Seoul, Republic of Korea) (once for 10 mg/kg) (CFAE) group, (3) a CFA + vehicle (same volume with normal saline with evogliptin tartrate/1 time) (CFAV) group, and (4) a CFA + indomethacin (Merk Korea, Seoul, Republic of Korea) (5 mg/mL/kg/1 time) (CFAI) group. The CFAE, CFAV, and CFAI groups were referred to as the CFA group. The experiment was conducted as follows (Figure 1).

In this study, the administered doses of evogliptin tartrate (10 mg/kg) were determined based on the results of previously conducted studies [12,13]. These studies administered a range of concentrations (1–100 mg/kg) orally or through diet in single or repeated treatments, effectively inhibiting plasma DPP-4. For our study, we selected a moderate dose of 10 mg/kg for repeated oral administration over a 5-day period. Additionally, we chose a body weight of 270 g to administer the drug during the experiment, which closely aligns with the typical body weight of 8-week-old animals, ensuring consistent drug dosing.

(a) On day 0 of the experiment, baseline measurements of plantar thickness and pain response to mechanical stimulation were taken. Subsequently, oral administration of drugs (evogliptin tartrate, vehicle, or indomethacin) took place, and 1 h later, inflammation was induced in 10 rats from each of the CFAE, CFAV, and CFAI groups. Inflammation was induced through an intraplantar subcutaneous injection of CFA (50%, 30 µL volume, prepared by mixing 1:1 saline to CFA [Sigma, #F5881]) into the left hind paw under anesthesia (isoflurane) (subcutaneous injection, 1 mL syringe, 26-gauge needle). For application of drug, in the CFAE group, evogliptin (DA-1229) tartrate (Suganon^®^, Seoul, Republic of Korea) was dissolved in saline and administered orally at a concentration of 10 mg/kg per dose. Administration was carried out once in the morning without anesthesia using a sonde throughout the experiment. The vehicle used for the CFAV group was saline, administered in mL/kg with the same volume as in the CFAE group. In the CFAI group, the NSAID indomethacin was administered. Indomethacin was dissolved completely in normal saline at the desired concentration; pH was adjusted to 7.4–8.0 using potassium hydroxide and hydrochloric acid. It was then suspended after shaking. Indomethacin was orally administered in the same manner as described above at a concentration of 5 mg/mL/kg once in the morning under non-anesthesia throughout the experiment.

(b) From day 1 to day 5, the drugs (evogliptin tartrate, vehicle, or indomethacin) were administered orally daily, and 1 h later, plantar thickness and pain response to mechanical stimulation were measured.

(c) After the behavioral experiments and plantar thickness measurements on day 5, inflamed plantar tissue and blood were extracted under anesthesia to determine cytokine levels (TNF-α and IL-1β) in the naïve, CFAE, and CFAV groups. Additionally, dorsal root ganglia (DRGs) were extracted for patch clamp experiments from the same groups. After collecting blood and tissue samples, all animals were promptly euthanized by cervical dislocation. The collected blood and tissue samples were homogenized and/or centrifuged to separate the supernatant and promptly frozen at −20 °C until use. Acutely cultured DRGs were promptly utilized for experiments and consumed within 12 h.

### 2.3. Measured Outcome

The rats were sacrificed after the 5-day treatment period, and blood samples were drawn from the heart while inflammatory tissue from rat plantar regions was collected to evaluate cytokines. The effect of evogliptin tartrate on managing inflammation and inflammatory pain was evaluated by measuring paw thickness of the rat plantar regions, paw withdrawal threshold, DRG resting membrane potential, DRG action potential firing, and cytokine (TNF-α and IL-1β) levels in plantar tissue and blood.

### 2.4. Measurement of Plantar Swelling

The injection of CFA into the sole elicited swelling by inducing an inflammatory response, and the degree of inflammation was determined by measuring the thickness between the sole and instep using a digimatic caliper (Bluetec, BD500, Seoul, Republic of Korea). The degree of anti-inflammatory activity was measured. Throughout the measurement of plantar swelling, group allocation was blinded.

### 2.5. Measurement of In Vivo Pain Response to Mechanical Stimulation

Pain responses were evaluated by measuring the paw withdrawal threshold (PWT) in response to von Frey filament stimulation (Touch Test Sensory Evaluators, North Coast Medical, Inc., Gilroy, CA, USA). Three days before the start of the experiment, the rats were allowed to adapt to the mesh floor and plastic cage for 2 h. On the day of the experiment, a 30 min to 1 h stabilization period was observed. A von Frey filament was applied to the center of the sole for 1–2 s, resulting in bending of the filament. The presence or absence of an avoidance response to the stimulus was then observed. A set of eight filaments (0.4, 0.6, 1.0, 2.0, 4.0, 6.0, 8.0, and 10.0 g) was used, and the test started with a 2.0 g filament. The PWT was measured and calculated using a simplified up–down method [14].

### 2.6. Measurement of Inflamed Plantar Tissue and Blood Cytokine Levels (ELISA)

After completing the plantar swelling and pain response tests on day 5, three rats were randomly selected from each group. The levels of TNF-α and IL-1β, which are representative inflammatory markers, were measured in plantar tissue and blood.

Inflamed plantar tissue was excised in a hexahedral shape from an area of approximately 2 × 2 cm in the central portion surrounded by the plantar process (six pads) to the plantar bone, including the skin and muscle. The tissue was placed in a radioimmunoprecipitation assay (RIPA) buffer and homogenized. After centrifugation at 13,000 revolutions per min for 10 min, the supernatant was aliquoted into a new tube and stored at −20 °C until the experiment.

From the selected rats, 2 mL of blood was extracted from the right atrium of the heart under respiratory anesthesia. The extracted blood samples were placed in tubes. The tubes were incubated in a water bath at 37 °C for 30 min, clotted, and then centrifuged at 3000× *g* for 15 min at 4 °C. The supernatant was aliquoted into a new tube and stored at −20 °C until the experiment.

The process of measuring cytokine levels using blood and inflammatory tissue samples was the same, and the ELISA kits used for measuring TNF-α and IL-1β were Rat TNF-α Immunoassay (Quantikine ELISA, #RTA00, R&D Systems, Minneapolis, MN, USA) and Rat IL-β/IL -1F2 Immunoassay (Quantikine ELISA, #RLB00, R&D Systems), respectively. The process was as follows: after removing the microplate strip, a foil was used to prevent light from entering; after adding 50 µL assay diluent to each well, 50 µL of standards and samples were added to each well and incubated at room temperature for 2 h; after removing samples from each well, the wells were washed with 400 µL of wash buffer; next, 100 µL of TNF-α and IL-1β conjugates were added to each well and incubated at room temperature for 2 h; the wells were washed again with 400 µL of wash buffer; after adding 100 µL of substrate solution to each well, they were incubated at room temperature for 30 min; and again, after adding 100 µL stop solution to each well, absorbance was measured at 450 nm using a microplate reader. The concentrations of TNF-α and IL-1β in each sample were measured based on standard results.

### 2.7. Patch Clamp Test Method in DRG Cells

As mentioned above, after completing the plantar swelling and pain response tests on day 5, six rats were randomly selected from each group. For each group, a patch clamp test was conducted using the current clamp method. Using this method, the cell body of the C-fiber within L4/5 DRG neurons, which primarily transmit pain signals, can be targeted. The minimum threshold value of electrical stimulation that generates the resting membrane potential and action potential of known small-sized DRG neurons (electrical capacitance ≤ 25 pF) was determined, and the number of action potentials generated by this electric stimulation was also recorded. The pain suppression effect of the drug was verified in vitro by comparing the excitability of the peripheral nervous system between normal pain models and the pain models treated with evogliptin tartrate.

Left L4 and L5 DRG were extracted from each selected sample and transferred to a phosphate-buffered saline (PBS) solution maintained at 4 °C. After removing the sheath and connective tissue, they were cut into small pieces and stirred for 45 min in 5 mL of modified Earles balanced salt solution (EBSS, pH 7.4) containing 0.7 mg/mL collagenase (type IA) and 0.3 mg/mL papain (35 °C, shaking water bath). After culturing, the nerve cells were separated by vigorous shaking, centrifuged at 1000 revolutions per min, and resuspended in DMEM (10% FBS + 1% penicillin/streptomycin). Neurons were transferred to cover glasses (poly-L-lysine coating; 12 mm; 01-115-20; Marienfeld-superior, Lauda-Königshofen, Germany) in a 24-well plate, and then incubated in a humidified incubator (95% O_2_, 5%) at 37 °C. All cells were used within 12 h of isolation.

Voltage fluctuations in the cell membrane were recorded using a current clamp with a patch clamp amplifier (MultiClamp 700A; Molecular Devices, San Jose, CA, USA). The measurement electrode was self-manufactured by pulling out a borosilicate glass capillary (#BF 150-86-10; Sutter Instrument, Novato, CA, USA) which had a resistance of 1.5–2.5 MΩ when the solution was filled inside the electrode. The plate containing nerve cells was observed with an inverted microscope (Model name: GX51, Olympus, Tokyo, Japan) and was perfused with extracellular fluid at the rate of 1–2 mL/min by gravity. The measuring electrode was attached to the cell membrane of the target cell, and negative pressure was applied to the cell membrane of the contact area to create a whole-cell state. The composition (mM) of the solution in the electrode was 140 mM KCl, 1.2 mM MgCl_2_, 4 mM MgATP, 0.4 mM Na2GTP, 10 mM hosphocreatine, 10 mM HEPES, and 0.5 mM EGTA (pH 7.2 with KOH, 298 mosm/kg H_2_O), and the extracellular perfusate (mM) was composed of 155 mM tetraethylammonium (TEA)-Cl, 2.5 mM CaCl_2_, 1.2 mM MgCl_2_, 14 mM glucose, and 10.5 mM HEPES (Ph 7.4 with TEA-OH, 320 mosm/kg H_2_O). The threshold at which an AP was generated was measured by injecting positive currents, gradually increasing from 0.1 to 1.2 nA for 2 ms, into the whole-cell configuration through a patch pipette solution. All reagents used in the experiments were purchased from Sigma-Aldrich, and the data were recorded and analyzed using pClamp software (Version 10, Molecular Devices).

Resting membrane voltage was measured within 10 s of switching to current-clamp mode after creating a whole-cell state using the membrane voltage clamping method. The electrical stimulation threshold required for AP generation was measured by increasing stimulation intensity from weak to strong, and the threshold value, which is the minimum electrical stimulation intensity required to generate an AP, was measured. The number of APs generated by electrical stimulation was measured as the number of action potentials generated by 50, 100, and 150 pA of electrical stimulation applied for 1 s.

### 2.8. Statistical Analysis

Data analysis was performed using the Prism 7 (GraphPad) program (GraphPad Software, San Diego, CA, USA). A two-way repeated-measures analysis of variance was conducted to compare repeatedly measured values of plantar thickness and pain response to mechanical stimulation between groups, and Sidak’s multiple comparison test was used for individual comparisons between groups at each specific time point. A one-way analysis of variance test was performed to compare the three groups of values measured at only one time point (cytokine level and patch clamp test results), and Tukey’s multiple comparison test was performed for individual comparisons between groups. It was judged that there was a statistically significant difference only when the *p* value was ≤0.05 (# or *, *p* < 0.05; ## or **, *p* < 0.01; ### or ***, *p* < 0.001).

## 3. Results

One rat in the CFAI group died before the experiment on day 5. Therefore, a total of 39 rats were included in this study.

### 3.1. Swelling of Inflammatory Plantar

**Figure 2:**
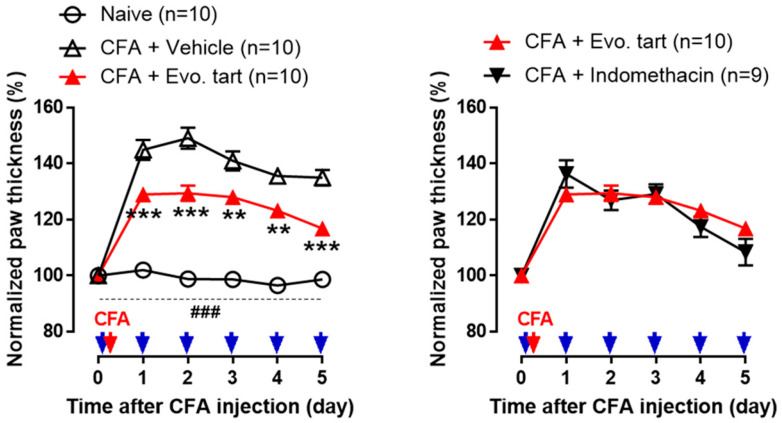
Comparison of paw thickness of the rats plantar region (CFA: complete Freund’s adjuvant, Evo. tart: evogliptin tartrate; ###: comparison between the naïve and CFAE groups; **, ***: comparison between CFAE group vs. CFAV group (**, *p* < 0.01; ### or ***, *p* < 0.001)) (red arrow: completion of CFA inflammation model, blue arrows: administration of drugs).

In the naïve group, no significant change in plantar thickness was observed until day 5, and it remained significantly lower than that in the CFA group (Figure 2). In contrast, in the CFA group, plantar paw thickness increased in all rats. In the CFAV group, plantar thickness significantly increased owing to the inflammatory response after CFA injection. In the CFAE group, plantar thickness significantly increased after CFA injection compared with the naïve group, but at the same time, it significantly decreased compared with the CFAV group, confirming the anti-inflammatory action of evogliptin tartrate. There was no significant difference between the CFAE and CFAI groups.

### 3.2. Pain Behavioral Test for Mechanical Stimuli

**Figure 3:**
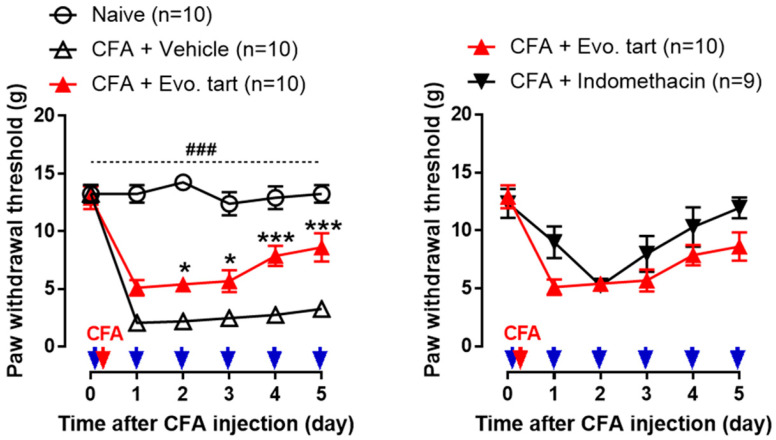
Comparison of avoidance response threshold values for von Frey filament stimulation (CFA: complete Freund’s adjuvant, Evo. tart: evogliptin tartrate; ###: comparison between the naïve and CFAE groups; *, ***: comparison between CFAE group vs. CFAV group (*, *p* < 0.05; ### or ***, *p* < 0.001)) (red arrow: completion of CFA inflammation model, blue arrows: administration of drugs).

In the naïve group, no change in PWT was observed until day 5, and PWT was significantly higher than that in the CFA groups (Figure 3). In the CFAV group, the PWT significantly decreased after CFA injection, indicating that hypersensitivity to mechanical stimuli was induced by an avoidance response to weak stimuli that generally do not induce pain. In the CFAE group, the PWT decreased after CFA injection; however, when compared with the CFAV group, it increased significantly from day 2, confirming that inflammatory pain was gradually alleviated. The analgesic action on this inflammatory pain was not significantly different from that in the CFAI group.

### 3.3. Resting Membrane Potential in Small-Sized DRG Cells

**Figure 4:**
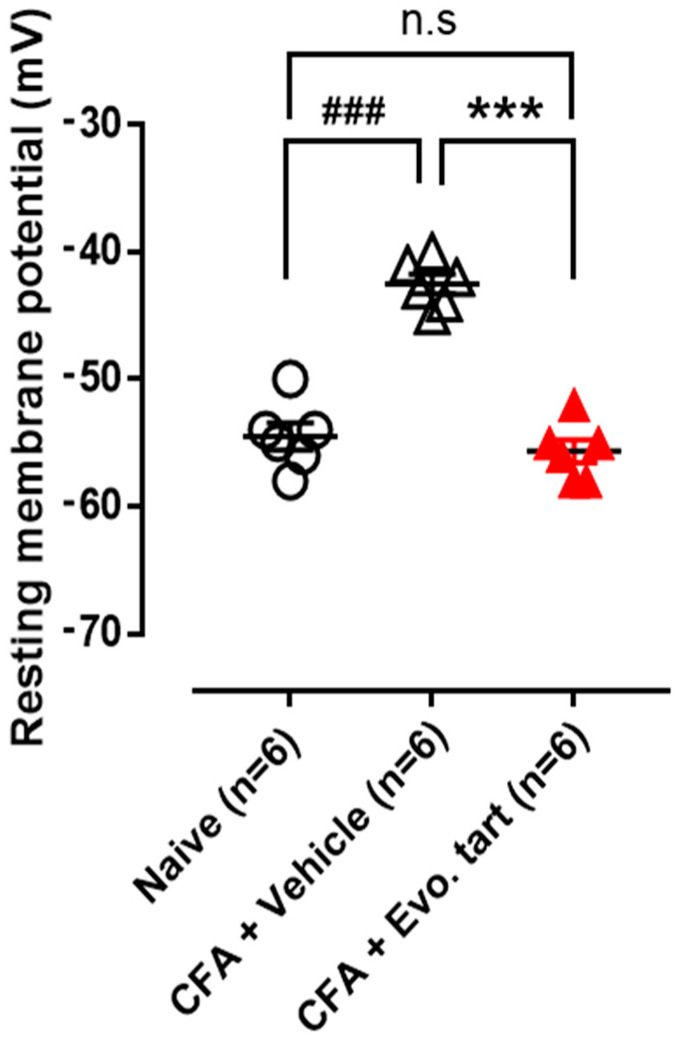
Resting membrane potential comparison (CFA: complete Freund’s adjuvant, Evo. tart: evogliptin tartrate, n.s.: not significantly different; ###: comparison between the naïve and CFAV groups; ***: comparison between CFAV group vs. CFAE group (### or ***, *p* < 0.001)).

The resting membrane potential of small-sized DRG cells in the CFAV group was higher (depolarized) than that in the naïve group (Figure 4), which meant that pain cells can spontaneously excite and generate pain signals (action potentials) even in the absence of stimulation. The abnormally depolarized resting membrane potential was normalized in the CFAE group.

### 3.4. Electrical Stimulation Threshold for Small-Sized DRG Cells

**Figure 5:**
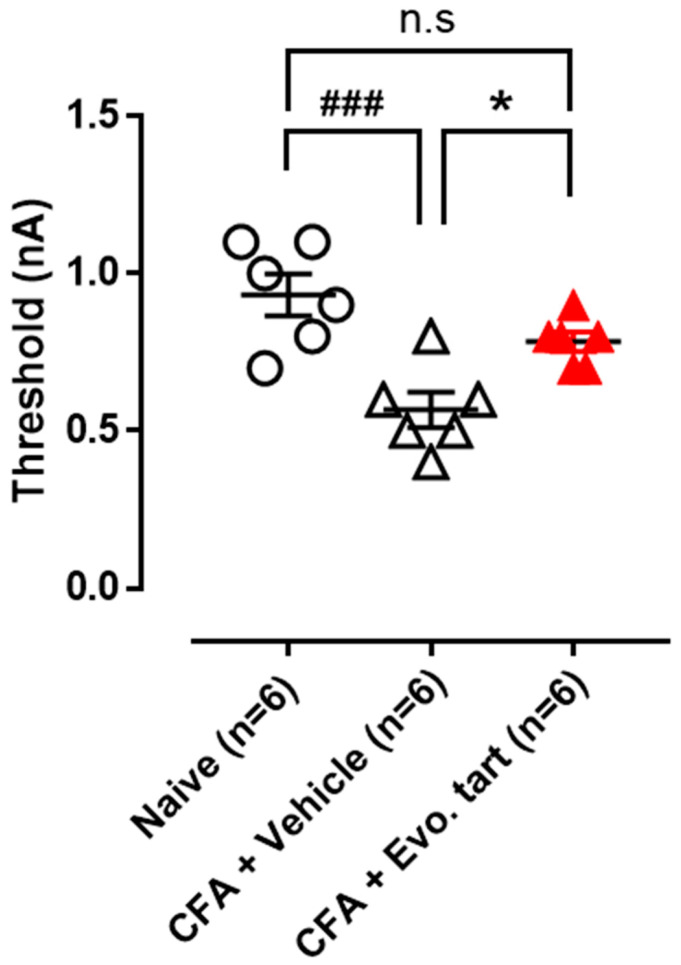
Minimum electrical stimulation threshold that generates an action potential (electrical stimulation threshold for small-sized dorsal root ganglion cells) (CFA: complete Freund’s adjuvant, Evo. tart: evogliptin tartrate, n.s.: not significantly different; ###: comparison between the naïve and CFAV groups; *: comparison between CFAV group vs. CFAE group (*, *p* < 0.05; ###, *p* < 0.001)).

**Figure 6:**
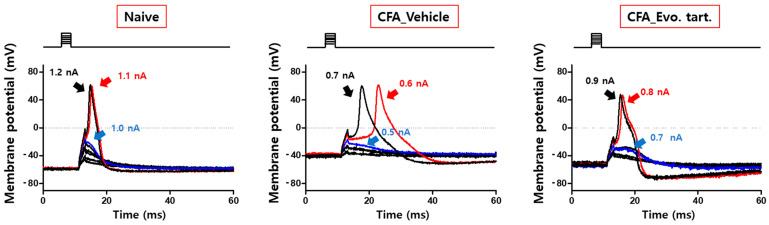
Electrical stimulation that induces an action potential for the first time (action potential firing in small-sized dorsal root ganglion cells) (CFA: complete Freund’s adjuvant, Evo. tart: evogliptin tartrate).

Electrical stimulation applied directly to DRG cells can generate an AP through membrane potential depolarization when it exceeds a certain level. Small-sized DRG cells in the CFAV group generated an AP even with electrical stimulation of lower intensity than that in normal cells (Figure 5). This implied that pain information can be generated even by small external stimuli that do not generally generate pain information. This abnormal decrease in the electrical stimulation threshold was recovered to a degree similar to that of the naïve group in the CFAE group. In Figure 6, the electrical stimulation that induced an AP for the first time in the naïve group was 1.1 nA; however, in the CFAV group, an AP could be induced at 0.6 nA. In the CFAE group, 0.8 nA was required to induce an AP.

### 3.5. Action Potential Firing in Small-Sized DRG Cells

**Figure 7:**
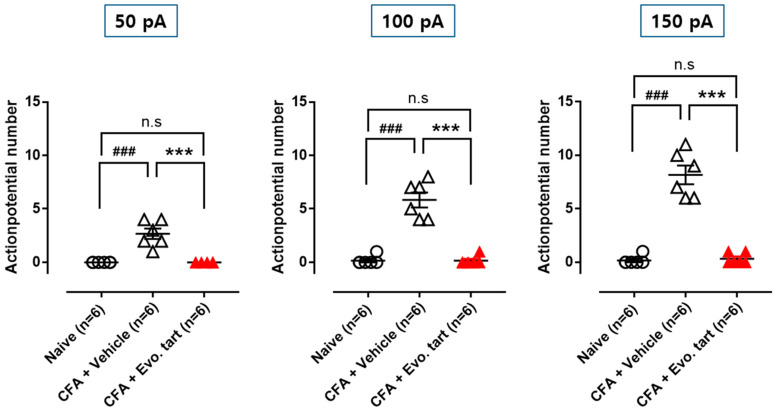
Number of action potentials evoked by the same electrical stimulation (CFA: complete Freund’s adjuvant, Evo. tart: evogliptin tartrate; n.s.: not significantly different; ###: comparison between the naïve and CFAV groups; ***: comparison between CFAV group vs. CFAE group (### or ***, *p* < 0.001)).

When electrical stimulation (50, 100, and 150 pA) of the same intensity was applied to the naïve, CFAV, and CFAE groups, small-sized DRG cells in the CFAV group generated more action potentials than those in the naïve group (Figure 7). This indicates that excessive pain information can be generated in the primary sensory nerves that respond to peripheral stimuli. This abnormal peripheral pain signal was normalized in the CFAE group.

### 3.6. Levels of TNF-α and IL-1β in Rat Paws and Serum

**Figure 8:**
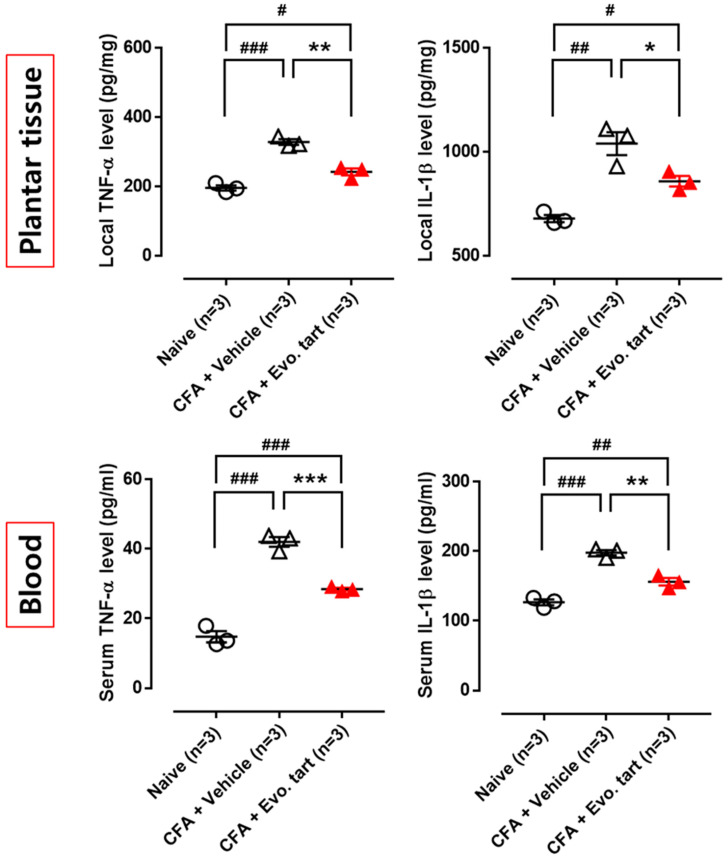
Cytokine levels in paw inflammation site and serum (CFA: complete Freund’s adjuvant, Evo. tart: evogliptin tartrate; n.s.: not significantly different; #, ##, ###: comparison between the naïve and CFAV groups; *, **, ***: comparison between CFAV group vs. CFAE group (# or *, *p* < 0.05; ## or **, *p* < 0.01; ### or ***, *p* < 0.001)).

Cytokine levels (TNF-α and IL-β) measured in the CFAE group were significantly higher than those measured in the naïve group in both inflamed paw tissue and serum (Figure 8). However, compared with the CFAV group, at the same time, cytokine levels were significantly reduced, confirming the anti-inflammatory action of evogliptin tartrate.

## 4. Discussion

Evogliptin tartrate, initially designed for managing blood glucose levels in patients with type 2 diabetes, operates by inhibiting the activity of DPP-4, thereby elevating the concentrations of incretin hormones such as GLP-1 and glucose-dependent insulinotropic polypeptide [15]. These hormones assume an important role in governing glucose metabolism, and emerging research has suggested their anti-inflammatory effects [16,17]. In the current study, we observed that evogliptin tartrate effectively diminished paw thickness, heightened the pain threshold, attenuated the transmission of pain signals within nociceptive nerves, and abated inflammatory cytokines in rats with inflammation and ensuing inflammatory pain, triggered by the intraplantar subcutaneous injection of CFA into the left hind paw. These findings indicate that evogliptin tartrate yields a favorable impact on curbing inflammation and alleviating pain resulting from inflammation.

Several studies have investigated the potential anti-inflammatory effects of evogliptin tartrate in various conditions [16,17]. Seo et al. suggested that treatment with evogliptin tartrate reduces the levels of inflammatory and fibrotic signaling in liver cells [17]. Furthermore, Razavi et al. found that evogliptin tartrate decreases DDP-4 and increases GLP-1 levels, consequently engendering metabolic changes capable of mitigating inflammation in patients with acute coronary syndrome [16]. Although the precise mechanism governing the anti-inflammatory effects of evogliptin tartrate is not yet comprehensively understood, these findings collectively imply the potential of the drug as a therapeutic intervention for conditions associated with inflammation. Nevertheless, further research is required to fully elucidate the anti-inflammatory properties of evogliptin tartrate and its potential therapeutic applications in clinical practice. This study aimed to confirm the possibility of expanding the indications for evogliptin tartrate use in patients with inflammatory pain.

Previous studies have demonstrated that GLP-1 analogs have modest anti-inflammatory effects by activating adenylate cyclase to produce cyclic adenosine monophosphate, which activates protein kinase A to activate the CAMP response element-binding protein [18,19,20,21,22]. Kang et al. reported that GLP-1 analogs inhibit interleukin-1β-induced inducible nitric oxide synthase at the protein level in RINm5F beta-cells [19]. Considering the ability of evogliptin tartrate to increase GLP-1 levels, we believe that its anti-inflammatory effects can be expected.

Painful stimuli are initially detected and received by peripheral nociceptive neurons and translated into APs. These APs are then conveyed along afferent neuronal pathways into the central nervous system, ultimately being interpreted as the sensation of pain [23]. Inflammatory reactions come into play during these stages, thereby giving rise to inflammatory pain. The outcomes of this investigation substantiated that evogliptin tartrate reinstated the resting membrane potential within the primary DRG in the CFA chronic inflammatory pain model by modulating the inflammatory response. Controlling the resting membrane potential is an important mechanism that regulates excitability [24,25]. Moreover, the minimum electrical stimulation intensity (threshold) required to generate an AP for transmitting a pain signal and the number of action potentials generated by electrical stimulation were restored to baseline levels. This suggests the potential to manage the abnormal transmission of pain stimuli through inflammatory reactions. In our in vivo behavioral experiment, the paw withdrawal threshold in the CFAE group was higher than that in the CFAV group. This trend mirrored the pattern observed in the indomethacin-treated group. This observation underscores that the anti-inflammatory effect of evogliptin tartrate closely parallels that of indomethacin—an agent extensively employed in clinical practice for mitigating inflammatory pain.

AP parameters in DRG neurons change during inflammation [26]. For example, augmentation of t-type calcium channel activity or a reduction in small-conductance calcium-activated potassium channels can decrease after-hyperpolarization (AHP) or increase AP duration and after-depolarization (ADP) in DRG neurons, contributing to the hyperexcitability of primary nociceptors in inflammatory and neuropathic pain [27,28,29]. Therefore, CFA-induced inflammation may increase the excitability of primary sensory neurons by modulating these two channels, contributing to inflammatory pain development. Furthermore, evogliptin tartrate reduces inflammation by inhibiting transforming growth factor-β signaling [17]. Therefore, evogliptin tartrate could ameliorate the hyperexcitability of DRG neurons in inflammatory pain by reducing AP duration and increasing AHP. Further studies are needed to investigate the detailed mechanism of evogliptin-induced inhibition of primary nociceptor hyperexcitability in pathological pain.

NSAIDs such as indomethacin encompass several potential side effects, including ulceration, bleeding, stroke, heart attack, dermatological changes, weight gain, edema, and breathing difficulties [30]. Patients experiencing such side effects necessitate alternative medications that can alleviate inflammatory pain through distinct anti-inflammatory mechanisms. Evogliptin tartrate presents itself as a plausible candidate for substitution.

## 5. Conclusions

In conclusion, evogliptin tartrate exhibited a favorable impact in diminishing inflammation and ameliorating inflammatory pain, akin to the effects of indomethacin. Furthermore, the hyperdepolarization of DRG cells incited by inflammation was rectified post administration of evogliptin tartrate. This paves the way for the potential expansion of the indications for evogliptin tartrate, either as an anti-inflammatory drug for various inflammatory diseases or as an analgesic in the context of inflammation-induced pain. However, our study was constrained by the small sample size of rats. Additionally, the effects of long-term evogliptin administration were not investigated. Additionally, the appropriate dosage of evogliptin tartrate for mitigating inflammation and inflammatory pain remains undetermined. Addressing these limitations warrants further investigation.

## Data Availability

The data presented in this study are available on request from the corresponding author.
